# Estimation of Combustion Parameters from Engine Vibrations Based on Discrete Wavelet Transform and Gradient Boosting

**DOI:** 10.3390/s22114235

**Published:** 2022-06-01

**Authors:** Achilles Kefalas, Andreas B. Ofner, Gerhard Pirker, Stefan Posch, Bernhard C. Geiger, Andreas Wimmer

**Affiliations:** 1 Institute of Thermodynamics and Sustainable Propulsion Systems, Graz University of Technology, 8010 Graz, Austria; andreas.wimmer@lec.tugraz.at; 2 Know-Center GmbH, Research Center for Data-Driven Business & Big Data Analytics, 8010 Graz, Austria; aofner@know-center.at (A.B.O.); bgeiger@know-center.at (B.C.G.); 3 LEC GmbH, Large Engine Competence Center, 8010 Graz, Austria; gerhard.pirker@lec.tugraz.at (G.P.); stefan.posch@lec.tugraz.at (S.P.)

**Keywords:** knock sensor, pressure sensor, virtual sensor, engine vibrations, combustion parameters, discrete wavelet transform, gradient boosting, explainable AI

## Abstract

An optimal control of the combustion process of an engine ensures lower emissions and fuel consumption plus high efficiencies. Combustion parameters such as the peak firing pressure (PFP) and the crank angle (CA) corresponding to 50% of mass fraction burned (MFB50) are essential for a closed-loop control strategy. These parameters are based on the measured in-cylinder pressure that is typically gained by intrusive pressure sensors (PSs). These are costly and their durability is uncertain. To overcome these issues, the potential of using a virtual sensor based on the vibration signals acquired by a knock sensor (KS) for control of the combustion process is investigated. The present work introduces a data-driven approach where a signal-processing technique, designated as discrete wavelet transform (DWT), will be used as the preprocessing step for extracting informative features to perform regression tasks of the selected combustion parameters with extreme gradient boosting (XGBoost) regression models. The presented methodology will be applied to data from two different spark-ignited, single cylinder gas engines. Finally, an analysis is obtained where the important features based on the model’s decisions are identified.

## 1. Introduction

Since the invention of the internal combustion engine, the in-cylinder pressure measurement was fundamental for the research and development of reciprocating engines on a global scale. Closed-loop control purposes, calibration, monitoring, diagnosis and validation of numerical modelling are tasks that depend on such measurements. A combustion analysis can be performed to reduce emissions and fuel consumption and improve performance based upon the in-cylinder pressure measurements [1]. Despite the accurate indication, these intrusive pressure sensors (PSs) have the disadvantages of high cost and issues with their durability [2,3,4]. In addition, the cylinder head requires modifications for the installation of these sensors, making the mounting process complex [1].

With the improved computational capability of the engine control units (ECUs), model-based replacement strategies for the in-cylinder PSs have become an interesting tool for engine control [5]. Empirical predefined approaches utilizing a Wiebe function for the determination of the mass fraction burned rate have been proposed [6]. These models require calibration for every engine operating condition.

The concept of virtual sensors can also be used to estimate combustion characteristics or even reconstruct the whole in-cylinder pressure from different source signals combined with various modelling approaches. In the study of Posch et al. [7], it was shown that a successful correlation build up was achieved with the use of the KS signals in addition to the intake manifold pressure measurements combined in a simple differential equation. In Wang et al. [8] such a virtual sensor was conceptualized by the use of extended Kalman filtering (EKF) and Frequency-Amplitude-Modulation Fourier series. Another approach was developed by Businaro et al. [9] in which it was shown that the first derivative of the in-cylinder PS is closely related to the vibration signal measured by a KS. A recursive in-cylinder pressure estimation method was implemented by Han et al. [10] which makes use of a Kalman filter and the vibration signal to gain information of the in-cylinder pressure. In Pla et al. [2], an extended Kalman filter is used to improve the estimation of the chosen physical models in order to receiving a more accurate estimation of in-cylinder pressure. Siano et al. [11] used a non-linear regression technique for the estimation of peak firing pressure (PFP) and its location with the filtered KS signal as input and the extraction of distinctive features. In recent years, machine learning approaches have been successfully applied for model predictive control of internal combustion engines, as shown in the study of Norouzi et al. [12]. Taglialatela et al. [13] used the crankshaft speed measurement as input for a multilayer perceptron network to receive the PFP and its location as output quantities. In Johnsson et al. [14], a reconstruction of in-cylinder pressure was performed based on complex radial basis function networks from vibration and speed signals. In Bennett et al. [15], a concept was proposed that makes use of measured crank angle (CA) kinematics as input for a recurrent neural network which approximates the in-cylinder pressure trace.

Discrete wavelet transform (DWT) is a multi-resolution decomposition and allows feature analysis associated with different frequency bands [16]. The received coefficients are resolved in the time and frequency domains and are therefore suitable for the analysis of non-stationary phenomena, as they are expected to be introduced by the complex combustion processes of an engine [17,18]. This signal-processing technique has proven to be effective in filtering the information of interest from overlayed noise [19]. In addition, the resulting coefficients of the DWT can be used to extract significant information, delivering a compact representation that can be used for further processing. Numerous gradient-boosting models have been applied successfully in many fields of research. They were implemented, for example, in the following areas: detecting intrusion attacks in wireless sensor networks [20]; fault detection in heat ventilation and air conditioning systems [21]; surface roughness prediction in high-speed milling in the metalworking industry [22]; structural damage classification in a wind-turbine foundation [23]; feature selection [24]; GPS signal reception classification [25]; and also for classification in predicting the interactions between target genes and drugs [26]. In the study of Nishat et al. [27], DWT was applied in combination with an XGBoost classifier for bearing fault detection of induction motors. The presented approach in this study consists of slicing the relevant part of the vibration signals acquired by a KS as input for the DWT with subsequent statistical feature extraction from the received coefficients. These features are then processed by the XGBoost models that perform various regression tasks to account for the chosen combustion parameters, which are the PFP in addition to the associated CAs at 10, 50 and 90% of the mass fraction burned (MFB10, MFB50, MFB90). The objectives of this research study are to find a convenient alternative for the in-cylinder PS that accomplishes an accurate estimation of the PFP and the MFB50. In addition, the capability of estimating the MFB10 and the MFB90 was investigated. For the fulfilment of tasks, a novel approach is presented which combines the DWT with an XGBoost regression model. In order to demonstrate the effectiveness of the proposed method, it was verified on comprehensive datasets from two different engines, proving to be robust to a change of engine block conditions.

This research article is organized in the following way: After this introduction in Section 1, the experimental work is presented in Section 2. Subsequently, Section 3 gives a detailed explanation of the developed method followed by Section 4 which reports the achieved results. After this, Section 5 will provide a discussion and finally Section 6 will present the conclusions.

## 2. Experimental Work

Experimental investigations were carried out on two spark-ignited single-cylinder research engines (SCE) with displacements of approximately 2.5 and 3 liters, operated in steady-state mode with natural gas. Multi-cylinder counterparts of these engines, with 12 to 20 cylinders, are used primarily for stationary power generation. Variations in ignition timing, excess air ratio and gas quality were performed as part of the single-cylinder engine tests, with measurements covering the entire range between misfiring and knocking to provide the broadest possible data base for developing simulation models for cycle-to-cycle variation and knocking. The two analyzed engines are designed with different pistons, a diverse bore and stroke of the cylinder, a varying compression ratio and ignition system. The connecting rod length only is preserved. For confidentiality reasons, the values for these characteristics cannot be presented.

**Figure 1:**
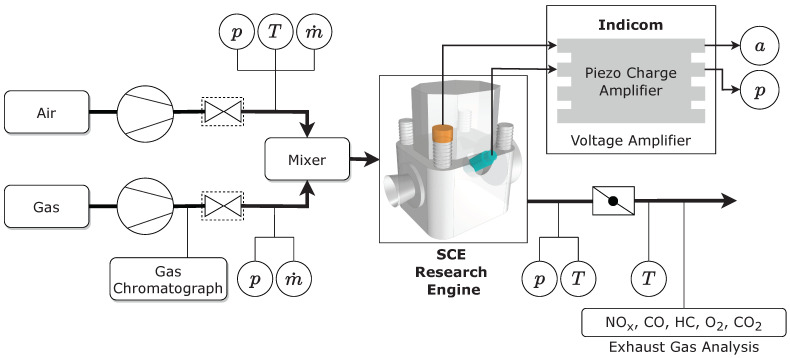
Schematic diagram of experimental setup with marked positions for in-cylinder PS (cyan) and KS (orange).

In Figure 1, a sketch of the exhaust side of such an SCE block is shown, where the position of the in-cylinder PS is shown in cyan color and the KS in orange color. Here, *p* denotes for the pressure, *T* stands for the temperature and $\dot{m}$ is the mass flow, while *a* denotes for the converted engine block vibrations measured by the KS. In addition, the schematic experimental setup is provided that shows the two main supply paths for air and gas, respectively. The air is conditioned by a complex control system to meet the required temperature, boost pressure and water content. The gas composition is determined through gas chromatography. Accurate mass flow measurements are achieved via Coriolis mass flow meters. Furthermore, sensors for temperature and pressure measurements are installed throughout the entire test bed. CA-resolved pressure measurements were generated for the intake and the exhaust manifold as well as for the combustion chamber. The exhaust path is equipped with a back pressure flap to imitate a turbine of a turbo-charger and a unit for exhaust gas analysis that determines the concentration of NOx, CO, HC, O_2_ and CO_2_ [28].

The in-cylinder PS applied in this study is the QC34C model of the AVL List GmbH. This intrusive, actively water-cooled PS works after the piezo-electrical principal where a directional deformation of the monocrystalline quartz crystals emits a charge that is converted by a charge amplifier to a voltage proportional to the acting force [29]. The signals coming from this sensor will be used to acquire the target information for the underlying regression tasks. In order to ensure the correct installation of the PS, an intrusive position is required that ideally is flush-mounted to the combustion chamber [1].

Parts of the vibrations of the engine block are caused by the combustion inside the combustion chamber and are recorded with a KS of type p/n 0 261 231 125 from Robert Bosch GmbH. This sensor is non-intrusive and works after the principle of a circular, seismic mass that counteracts the vibrations due to inertia. This mass interacts with a fixed piezo-crystalline layer that emits a specific charge in accordance with the impact force. The charge is converted by a charge amplifier to a voltage proportional to the intensity of the vibrations [29]. The positioning of these KSs is crucial and can deliver different signals with varying signal quality. In serial applications, which consist of multiple single cylinder engines, a KS is positioned on one of the main bolts for each cylinder head. This position is parallel to the axis of the cylinder and has a certain engine block transfer function from the combustion chamber to the KS, as shown in Figure 1. Among the advantages of this position is that it is common for most of the engines comparable to the ones investigated, since the mechanical concept comes with four large dimensioned bolts. No additional machining step for mounting of the KSs on these bolts is necessary due to the surface being already prepared with an appropriate drill included.

**Figure 2:**
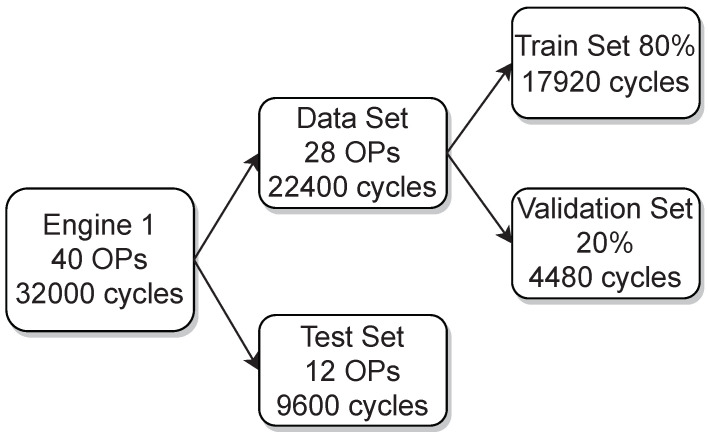
Datasets and performed splits of engine 1.

**Figure 3:**
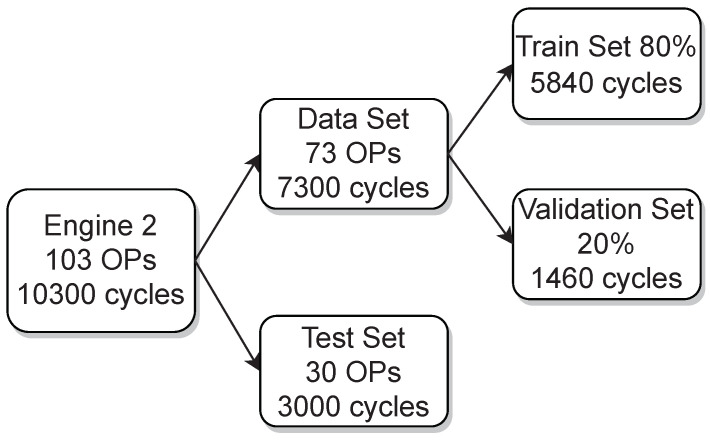
Datasets and performed splits of engine 2.

**Figure 4:**
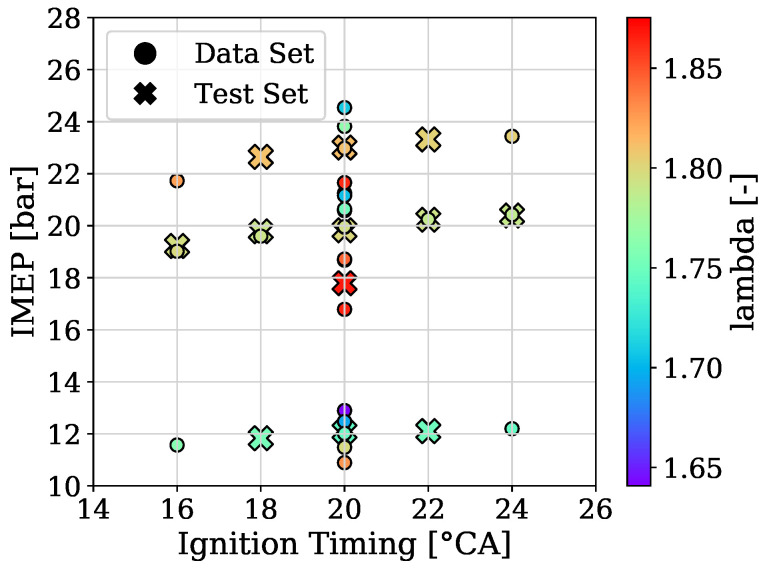
OPs of engine 1.

**Figure 5:**
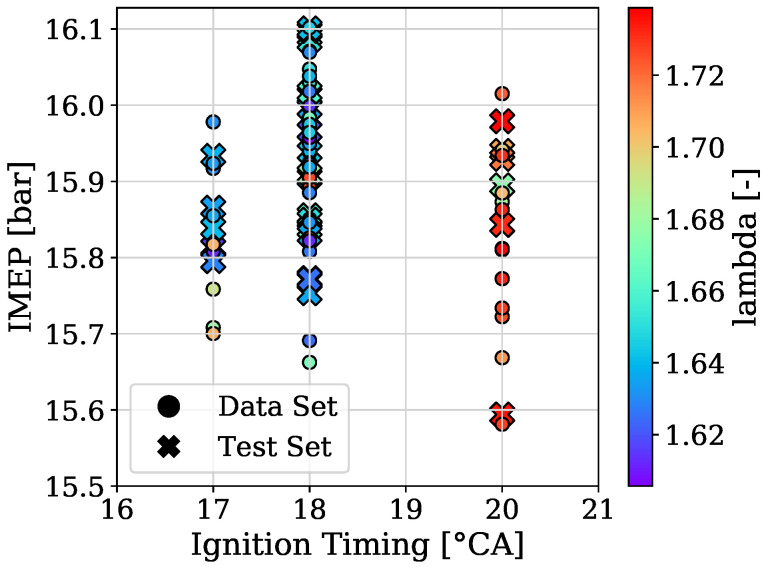
OPs of engine 2.

To provide a picture of how the acquired data of engine 1 and engine 2 was split for training, validation and testing Figure 2 and Figure 3 are presented, respectively. In addition, the variation of the three parameters from the used OPs, which are the ignition timing, the indicated mean effective pressure and the air–fuel equivalence ratio (lambda), are shown in Figure 4 for engine 1 and in Figure 5 for engine 2.

## 3. Methods

**Figure 6:**
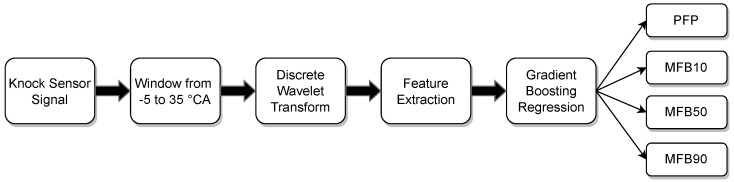
Overview of introduced method including regression targets.

A cycle of a four-stroke reciprocating internal combustion engine is constituted by the intake, the compression, the power (combustion) and the exhaust stroke. Since the combustion process is of interest for the present study, a major portion of the relevant stroke was analysed. With the presented method, cyclic estimations of combustion parameters were gained only by the use of vibration data coming from a KS. This approach consists of four major steps: (i) slicing the part of the KS signal that contains the relevant power stroke information for estimating the combustion parameters closely related to the in-cylinder pressure trace; (ii) applying DWT on the window from the signal, receiving levels of approximation coefficients (AC) and detailed coefficients (DC), from which; (iii) a set of defined statistical features is extracted; (iv) these features will serve as the input for the XGBoost regression models. Figure 6 gives an overview of the presented method.

As a comparison method, all regression experiments were performed additionally by extracting the same statistical features from the time and the frequency domains of the sliced signals. These obtained features served as input for the XGBoost models.

### 3.1. Combustion Parameters

Various combustion parameters have been introduced depending on the type of engine, the combustion modes and the injection strategies. In this study the PFP, the MFB10, MFB50 and MFB90 were selected. These quantities are conventionally computed from the in-cylinder pressure and are utilized as targets for the regression where the only input is the KS signal.

The monitoring of PFP is important due to its function as a critical structural constraint [1]. In addition, this information can be used for balancing out all cylinders of multi-cylinder engines. The MFB values provide a measure for the fraction of energy released from the combustion of fuel to the total energy released at the end of the combustion process [30]. The MFB50 parameter in particular provides valuable insights if an optimal combustion phasing occurs [31,32]. Furthermore, the difference of the MFB90 and the MFB10 gives an estimation of the burn duration. In general, the estimation of these MFB values is based on the calculation of the apparent heat release from the measured in-cylinder pressure using the first law of thermodynamics with single-zone assumption. This apparent heat release $\frac{\partial Q}{\partial \phi }$ with neglected wall heat transfer and blowby losses can be derived as [1]:

(1) $$\frac{\partial Q}{\partial \phi }=\frac{\gamma }{\gamma -1}p\frac{dV}{d\phi }+\frac{1}{\gamma -1}V\frac{\partial p}{\partial \phi }$$

In the above Equation (1), γ denotes the heat capacity ratio and is chosen as constant value of 1.32 with respect to the occurring gas temperatures. The volume *V* of the cylinder is derived by the engine law of kinematics [1]. From $\frac{\partial Q}{\partial \phi }$ the normalized cumulative sum can be calculated and the MFB values at the desired percentage can be extracted.

### 3.2. Sensor Signals Window

**Figure 7:**
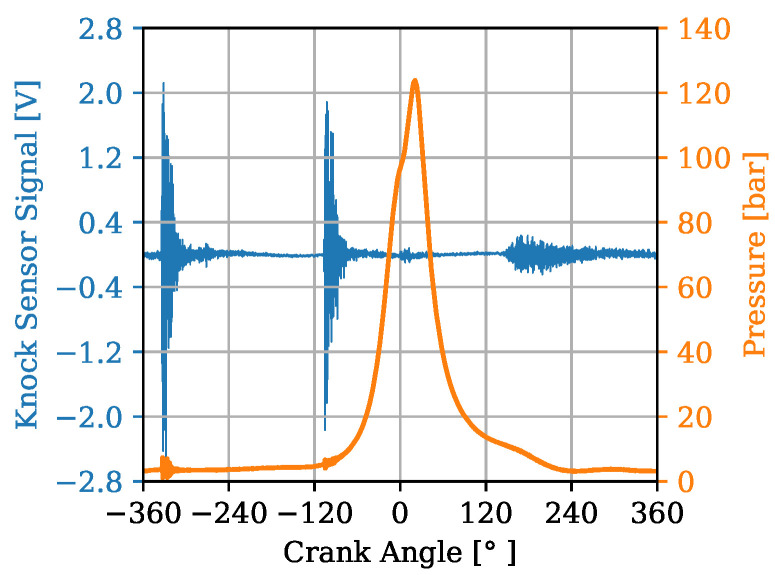
Whole cycle signals from −360 to 360 °CA.

**Figure 8:**
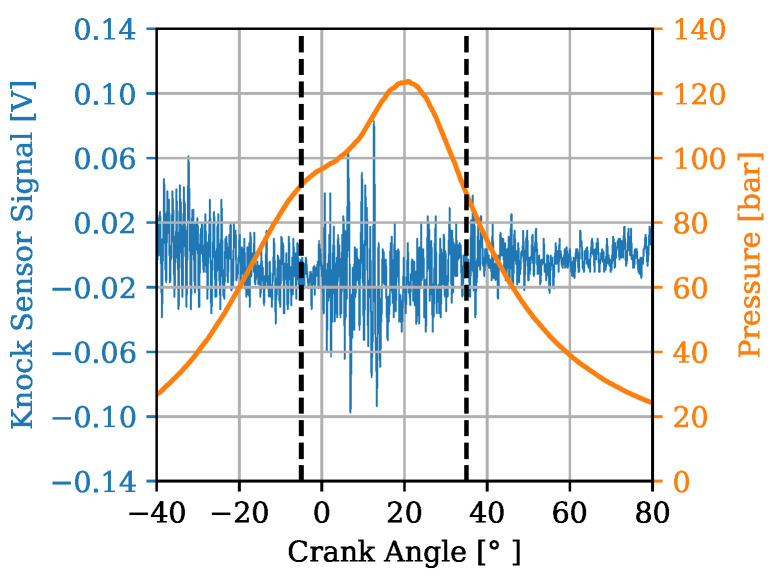
Window of cycle signals from −5 to 35 °CA.

In Figure 7 and Figure 8, an example of a cycle is shown where the KS signal in presented in blue with the corresponding axis on the left. In addition, the in-cylinder pressure is plotted in orange with its associated axis on the right. The time range of the signals recorded from the KSs is from −360 to 360 °CA. Since the goal is to receive accurate estimations of combustion parameters it must be ensured that a relevant window slice is taken from the combustion stroke of the cycles. Due to a transfer function of the engine block from the combustion chamber to the KS a possible delay in the signals has to be taken into account. Diverging from the study of [11], in which a window was chosen ranging from 20 to 80 °CA, a window ranging from −5 to 35 °CA was selected here. This is in accordance with to the findings of [9]. It is of great importance to select an appropriate window of the signals that depicts the phenomena of interest and excludes irrelevant ones. Therefore, this choice is designed to exclude, for instance, the prominent clapping noise from the intake and the exhaust valves which can be observed by looking at the high-amplitude regions of the blue signal on Figure 7. A major portion of the combustion phase with regard to the PFP is included in the window slice shown on Figure 8.

### 3.3. Discrete Wavelet Transform and Feature Extraction

The main idea of discrete wavelet transform is the decomposition of a given signal into a number of levels, where each level is a time series of coefficients describing the evolution of the signal in corresponding frequency bands [33]. There are two kinds of wavelet transforms: the continuous and the discrete one.

The continuous wavelet transform (CWT) of a signal p(ϕ) with respect to the wavelet function Ψ(ϕ) is defined as [34]:

(2) $$T\left(a,b\right)=w\left(a\right)\int_{-\infty }^{\infty }p\left(\phi \right)\psi^{*}\left(\frac{\phi -b}{a}\right)d\phi$$

where ∗ denotes complex conjugation. The term w(a) is typically set to $1/\sqrt{a}$ for reasons of energy conservation. Thus, the normalized wavelet function is often written more compactly as:

(3) $$\Psi_{a,b}\left(\phi \right)=\frac{1}{\sqrt{a}}\psi \left(\frac{\phi -b}{a}\right)$$

As will be shown, the DWT accounts for less computational expense since infinite summations of discrete wavelet coefficients can be used, rather than continuous integrals as required for the CWT. With logarithmic discretization it is possible to link the scale *a* to the size of steps taken between *b* locations in a natural way. This discretization of the wavelet has the following form, where integers *m* and *n* control the wavelets’ dilation and translation respectively [35]:

(4) $$\Psi_{m,n}\left(\phi \right)=\frac{1}{\sqrt{{a_{0}}^{m}}}\psi \left(\frac{\phi -nb_{0}{a_{0}}^{m}}{{a_{0}}^{m}}\right)$$

In Equation (4), $a_{0}$ stands for a specified fixed dilation step parameter set at a value greater than 1, whereas $b_{0}$ is the location parameter greater than 0. The wavelet transform of the continuous signal p(ϕ), using discrete wavelets as defined above is [35]:

(5) $$T_{m,n}=\int_{-\infty }^{\infty }p\left(\phi \right)\frac{1}{{a_{0}}^{m/2}}\psi \left({a_{0}}^{-m}\phi -nb_{0}\right)d\phi$$

With the derived wavelet transform of Equation (5) a simple reconstruction formula for the signal p(ϕ) is obtained by the infinite series as [35]:

(6) $$p\left(\phi \right)=\sum_{m=-\infty }^{\infty }\sum_{n=-\infty }^{\infty }T_{m,n}\psi_{m,n}\left(\phi \right)$$

Since combustion events introduce non-stationary effects within the signals recorded by the KS, DWT is more suitable than classic Fourier analysis due to revealed information in the time and frequency domain [16]. In initial experiments, several mother wavelets were tested and it was discovered that, for the present experimental settings, the Haar wavelets performed best. This claim is confirmed by the PFP regression results shown on Table 1 where, according to [27], four widely used mother wavelets were applied. The selected evaluation metrics for the results of this study are: (i) the root mean squared error (RMSE) which is defined as $\sqrt{\frac{1}{n}\sum_{i=1}^{n}{\left(y_{i}-\hat{y_{i}}\right)}^{2}}$; (ii) the mean absolute error (MAE) given as $\frac{1}{n}\sum_{i=1}^{n}\left|y_{i}-\hat{y_{i}}\right|$; and (iii) the coefficient of determination ($R^{2}$) derived as $1-\frac{\sum_{i=1}^{n}{\left(y_{i}-\hat{y_{i}}\right)}^{2}}{\sum_{i=1}^{n}{\left(y_{i}-\bar{y_{i}}\right)}^{2}}$.

**Figure 9:**
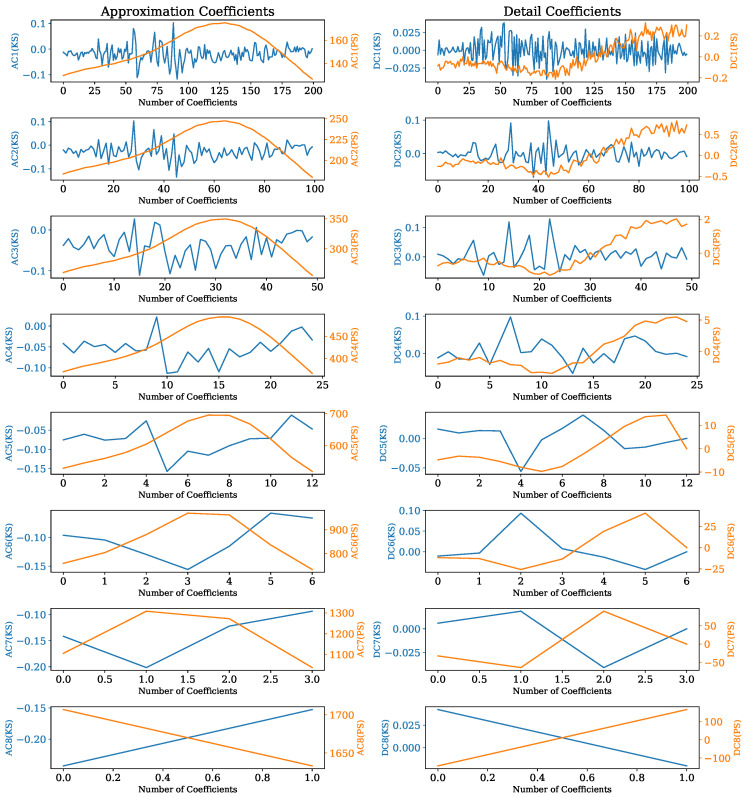
Resulting AC and DC from DWT of signals window.

The number of levels obtained from DWT can be calculated as $log_{2}\left(samples\right)$ [36]. A sampling frequency of $f_{s}=90$ kHz is derived from the rotational speed of 1500 rpm and the sampling rate of 0.1 °CA. For the selected window of the KS signal a(ϕ), 400 samples are received, which yields eight levels. By applying the above-introduced DWT to the arbitrarily chosen cycle of Figure 8, the blue coefficients are received from the KS signals with the associated blue axis values on the left, whereas the orange signals shown are acquired by the in-cylinder PS signals with the corresponding orange axis values on the right of each subplot in Figure 9. The approximation coefficients (AC) constitute a filter where mainly low-frequency portions are passed, whereas the detailed coefficients (DC) represent the high-frequency counterpart.

**Figure 10:**
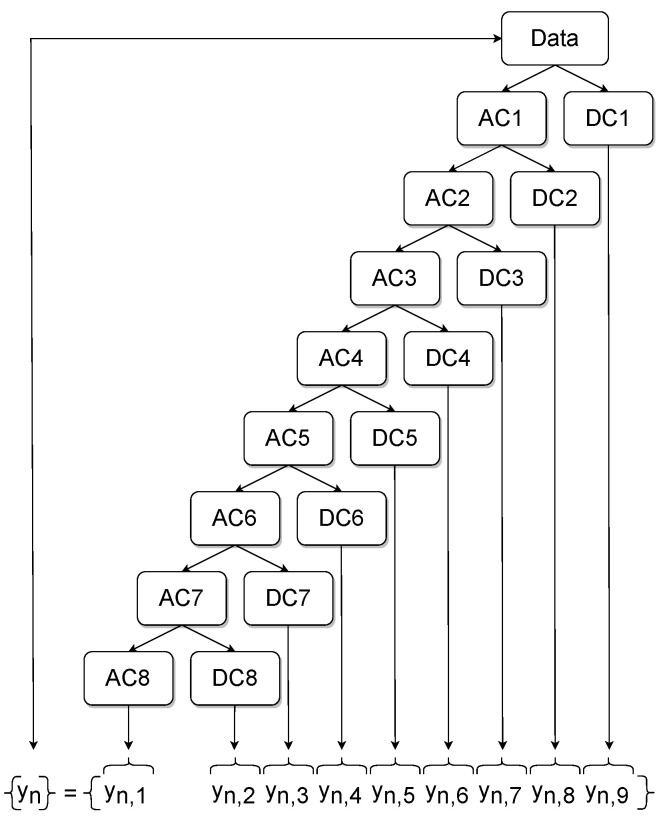
Feature Extraction from AC and DC of DWT Levels.

In this study, various statistical features that were proposed by [27,37] were utilized for the present regression tasks, but only few of them proved to be necessary for obtaining a precise estimation of the desired combustion parameters with regard to the KS signals a(ϕ). Thus, the obtained features presented in Table 2 were extracted from every set of DC from the corresponding DWT level. Additionally an extraction of these features was performed from the AC of DWT level eight accounting for the low frequency basis. An illustration of this process is given in Figure 10. Finally, an array of 8×9=72 features was obtained for every individual cycle. This extracted information served as input for the XGBoost regression algorithm.

### 3.4. Extreme Gradient Boosting (XGBoost) Regression and Feature Importance (FI)

Gradient Boosting is categorized as a so-called ensemble method, which refers to models employing multiple learning algorithms at the same time. Usually these models are decision trees that build on the principle of ensemble learning, where multiple weak learners collaborate to produce a model that performs better [20,24]. In particular cases, further developed gradient boosting implementations such as XGBoost may produce trained models that reach higher accuracy than artificial neural networks (ANNs) and ordinary least square regression models [38]. The computational complexity of these models is reduced by an automatic learning of the nodes’ splitting process, as well as a good performance in distributed computation [20]. Furthermore, the problem of overfitting was addressed by appending a regularization term Ω(θ) to the objective function within the learning procedure [39], as given in Equation (7).

(7) obj(θ)=L(θ)+Ω(θ)

In Equation (7), θ denotes the best set of parameters, whereas L(θ) is the training loss function and was defined as the commonly used mean squared error which is given in Equation (8) where $y_{i}$ are the labels and $\hat{y_{i}}$ the predictions of the model.

(8) $$L\left(\theta \right)=\sum_{i}{\left(y_{i}-\hat{y_{i}}\right)}^{2}$$

The regularization term is defined as Equation (9).

(9) $$\Omega \left(\theta \right)=\sum_{k}\Omega \left(f_{k}\right)$$

Each independent tree structure and leaf weights corresponds to a $f_{k}$ and is therefore taken into account by the regularization term Ω.

One advantage in comparison to ANNs is that the approach is simpler, meaning the tuning of hyperparameters and training is fast and can be easily performed by grid search and k-fold cross validation. In general, the amount of data required for successful training can be smaller compared to ANNs. Furthermore, the computationally efficient implementation of XGBoost makes such an approach lucrative. By utilizing a grid search on the present training sets and choosing a 5-fold cross validation, a set of optimized hyperparameters was obtained. These are: a maximum depth of five; a learning rate of 0.1; 5000 estimators; and a subsample of 0.8. To prevent overfitting, an early stopping was applied by choosing 100 rounds.

An improved understanding of the decision-making mechanism of an ML model is an important task to ensure fairness, robustness and causality while attaining the best prediction [40]. In the present study, two techniques were applied to provide a picture where the useful information is located. The first one is the FI based on mean decrease in impurity (MDI), which is defined as the total decrease in node impurity averaged over all trees of the ensemble [41]. For every feature *n* the decrease in impurity is the sum of the split gain that uses this feature. This split gain constitutes the amount of decrease in impurity with regard to the split [40]. The results obtained from this analysis are colored and stacked bar plots that show the distribution of FI according to the MDI across the selected features. The second technique is based on the Shapley additive explanations (SHAP). These SHAP values are calculated by introducing the features, one at a time, into a conditional expectation function of the model’s output and attributing the change produced at each step to the feature that was introduced. Finally, an averaging of this process over all possible feature orderings is performed. This measure has proven to be accurate and consistent [42].

## 4. Results

### 4.1. PFP Regression and FI

**Figure 11:**
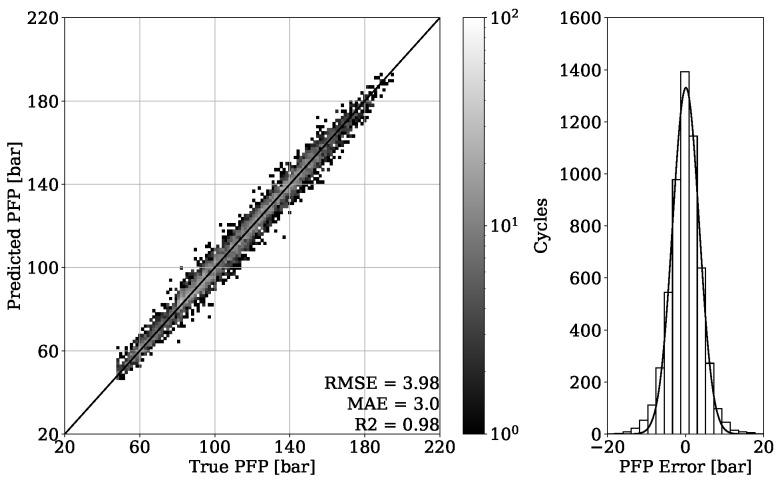
PFP regression of validation set from engine 1.

**Figure 12:**
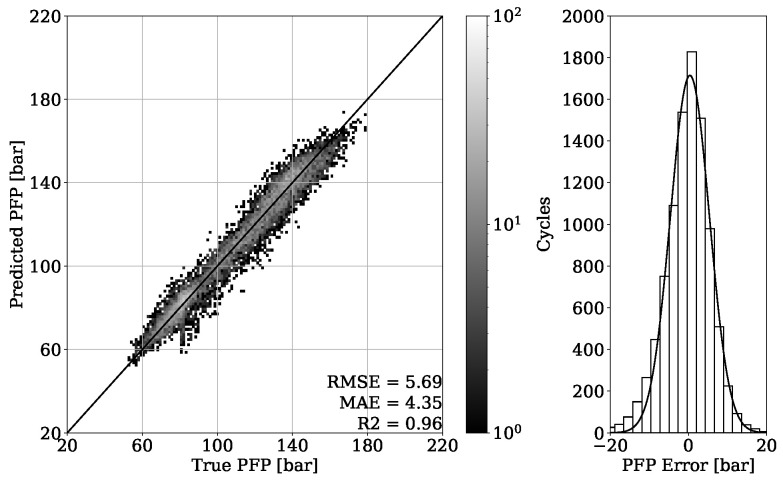
PFP regression of test set from engine 1.

In Figure 11, the regression result from validation set of engine 1 is presented. It can be seen that the obtained model is able to accurately estimate the in-cylinder PFP from engine vibrations, which is proven by achieving a MAE of 3.0 bar. The error distribution of Figure 11 shows a Gaussian characteristic with the mean error value around zero, indicating no systematic error. To be able to evaluate the model generalization properties, different operating conditions are tested, as shown in Figure 4. The MAE of 4.35 bar given in Figure 12 is slightly higher compared to the validation set. Nevertheless, the major concentration of cycles, which is indicated by the light-gray color, is very close to the perfect fit. This can also be observed by looking at the error distribution diagram of Figure 12.

**Figure 13:**
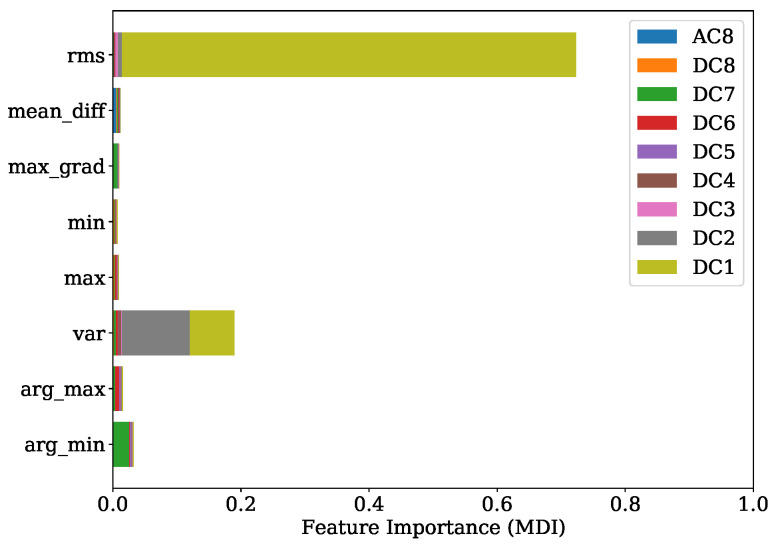
FI of PFP regression with regard to training set of engine 1.

**Figure 14:**
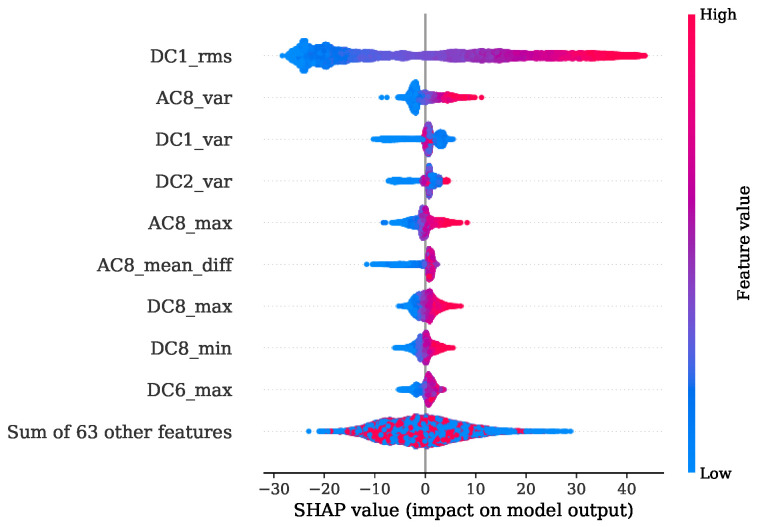
SHAP values summary of PFP regression with regard to training set of engine 1.

Figure 13 shows the FI values of the training procedure as a stacked histogram in which the designated colors represent the corresponding DC of all DWT levels, including the AC of level 8. The root mean squared (RMS) feature of level 1 contributes by far the most to this. The second most informative features are the variances from level two and level one. In comparison to this evaluation a summary representation of the SHAP values is presented in Figure 14 as a bee swarm diagram, summarizing various impacts of the model output. Here, every cycle analyzed is represented by a dot. On the x-axis, the SHAP values are given, whereas the y-axis shows a list of the nine most-contributing features resulting from this analysis. An associated colormap marks the feature values, where the magenta color stands for a high value in contrast to the blue color. It is shown that this method is in good accordance with the FI analysis and finds the variance of the AC from level eight as additional important contribution. Note that the point cloud of the remaining 63 features is disarranged in color.

**Figure 15:**
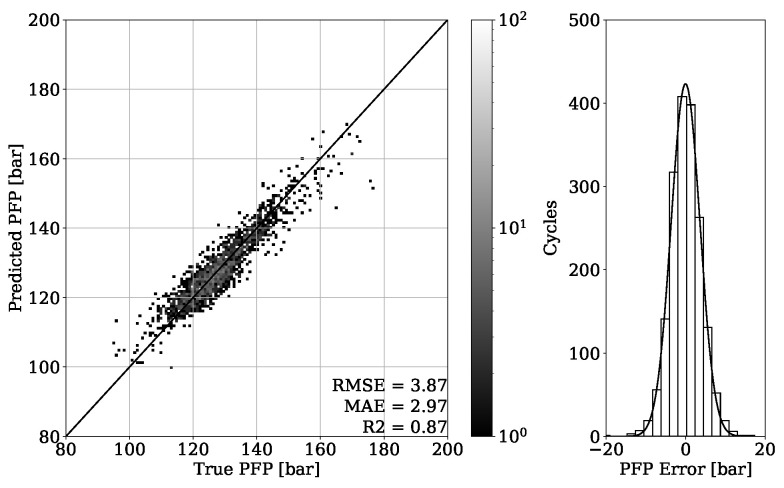
PFP regression of validation set from engine 2.

**Figure 16:**
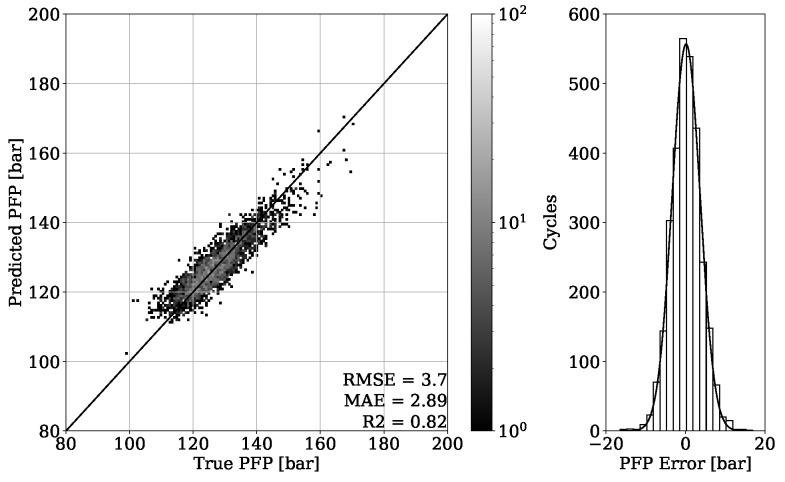
PFP regression of test set from engine 2.

Looking at the results from the regression of the PFP of engine two data presented on Figure 15, a similar impression compared to the previous analysis of engine one data is obtained. The MAE of 2.97 bar once again shows an accurate capability of estimating the PFP from the validation set. In addition, the error distribution confirms a Gaussian characteristic centered around zero with no systematic error occurring. Figure 16 shows the results obtained from the test dataset of engine 2. By achieving an MAE of 2.89 bar, the generalization property of the model is proven.

**Figure 17:**
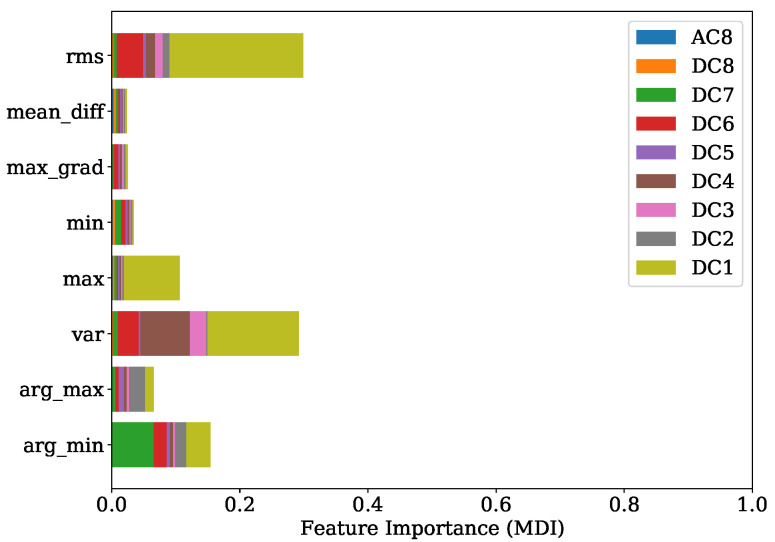
FI of PFP regression with regard to training set of engine 2.

**Figure 18:**
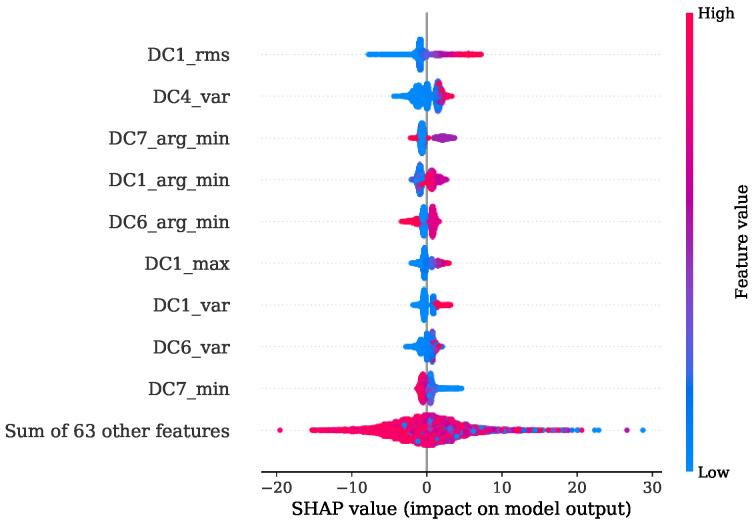
SHAP values summary of PFP regression with regard to training set of engine 2.

Figure 17 shows the FI based on MDI. It can be seen that the RMS and variance together with the location of minimum are the most prominent features. Features coming from levels 1, 4, 6 and level 7 in particular are ranked as important. The overview obtained from the SHAP representation of Figure 18 is in good accordance to the findings of the FI based on MDI. The results show that most of the selected features contribute to achieve a better performance. This is also indicated by the distinct magenta point cloud of the remaining 63 features.

### 4.2. MFB50 Regression and FI

**Figure 19:**
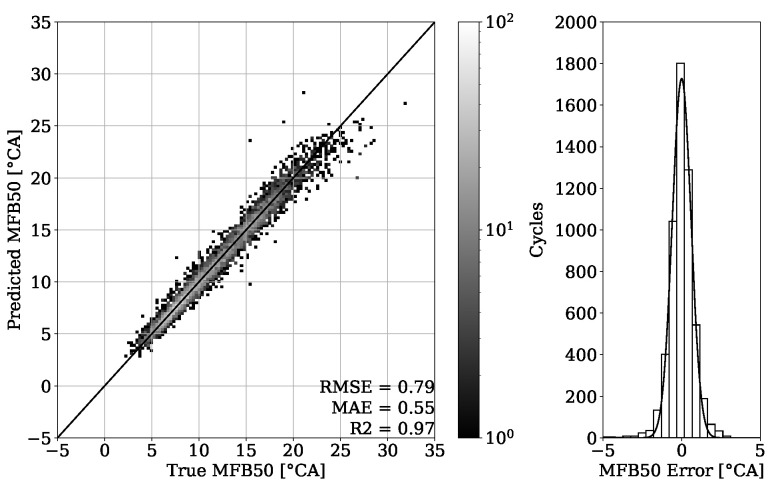
MFB50 regression of validation set from engine 1.

**Figure 20:**
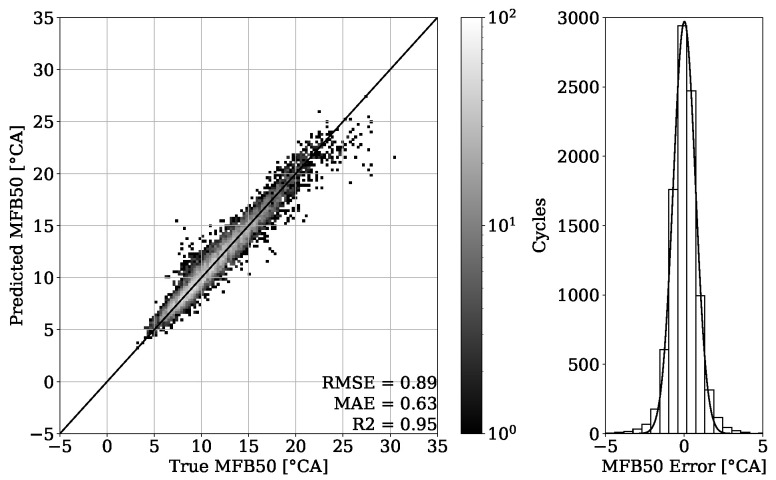
MFB50 regression of test set from engine 1.

The results of combustion phasing parameter MFB50 on Figure 19 and Figure 20 show an accurate estimation capability of the presented approach. The MAE for the validation set of engine 1 is 0.55 °CA with no systematic error occurring and the MAE of the test set experiment from the same engine is 0.63 °CA. Besides this good general performance, it can be noted that at higher MFB50 values, individual cycle events tend to be further away from the perfect fit. This can be explained by a late combustion process, which has usually a flat pressure curve characteristic with no distinct peak. Thus, the resulting small gradients can make a correct estimation of the MFB50 more challenging. Since this is a condition that to this extent occurs only occasionally, these particular cycles are poorly represented within the dataset.

**Figure 21:**
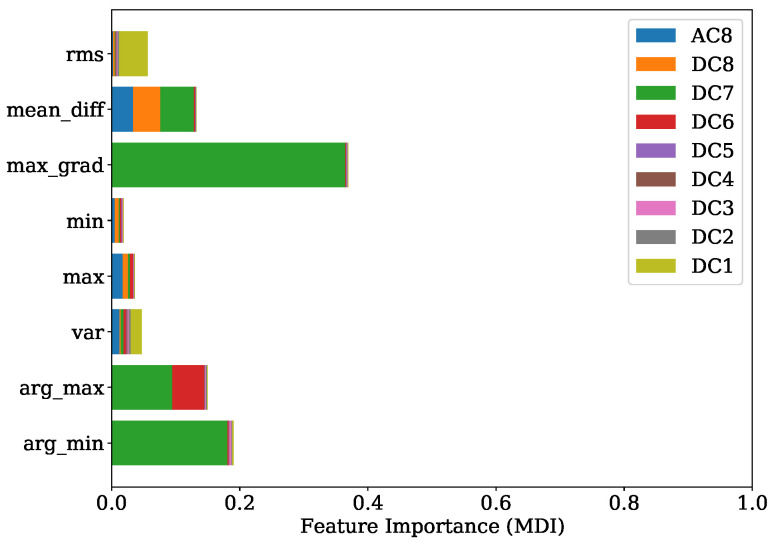
FI of MFB50 regression with regard to training set of engine 1.

**Figure 22:**
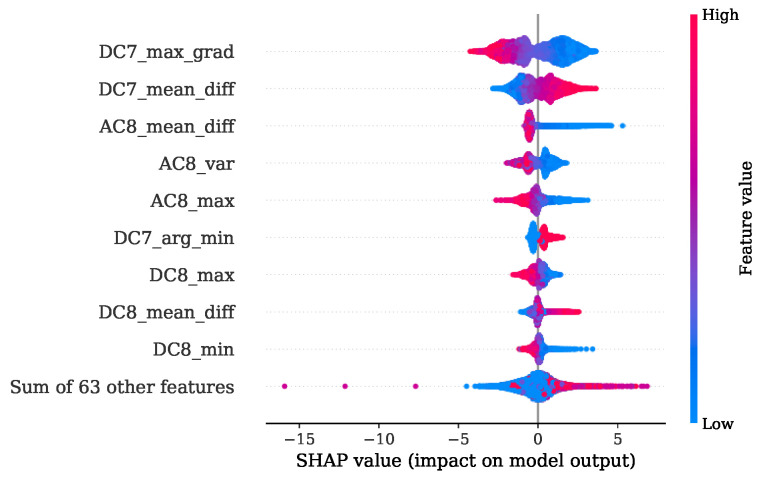
SHAP values summary of MFB50 regression with regard to training set of engine 1.

Once more, a good agreement is obtained of the top-ranked features received from FI based on MDI analysis given in Figure 21 and the SHAP evaluation presented by Figure 22. Here, the maximum gradient feature, the mean difference and the location of minimum and maximum are decisive. In particular, level 7 is of primary importance. Level 8 features are additionally found to be contributing, as shown by the SHAP beeswarm diagram.

**Figure 23:**
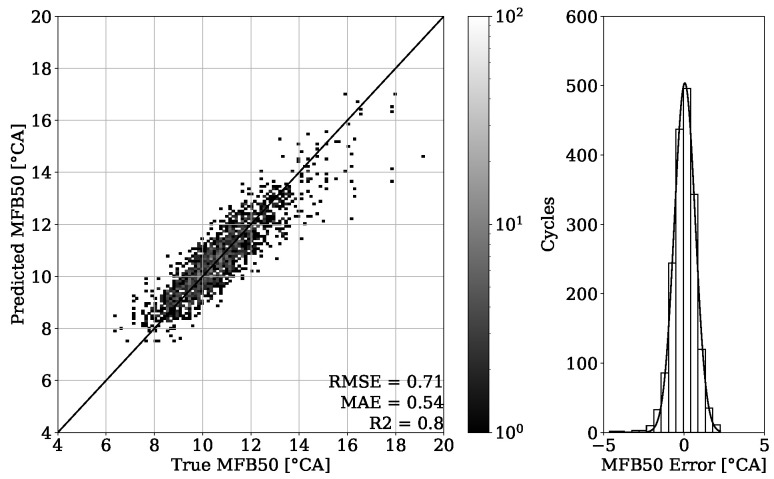
MFB50 regression of validation set from engine 2.

**Figure 24:**
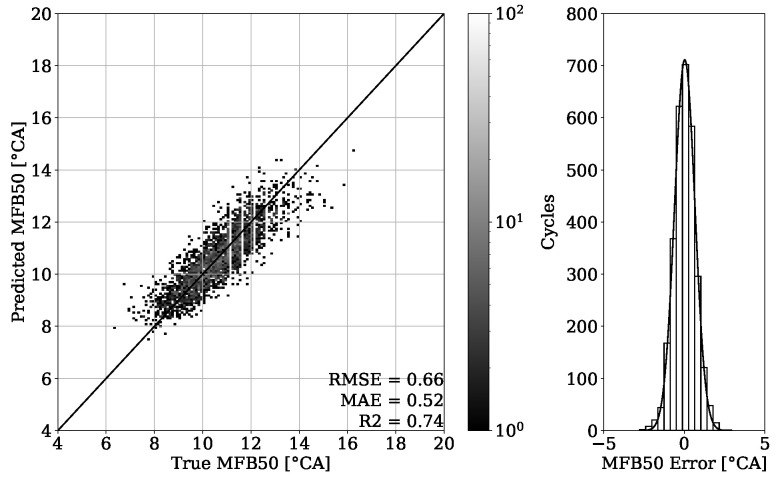
MFB50 regression of test set from engine 2.

The results of Figure 23 also show a convincing performance for engine 2 with the MAE value of 0.54 °CA. By testing the model on different operating conditions as shown in Section 2, an MAE of 0.52 °CA could be achieved (Figure 24).

**Figure 25:**
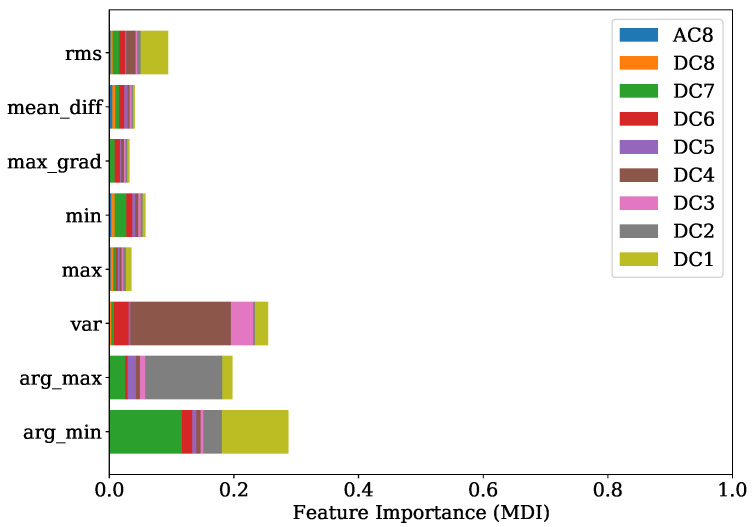
FI of MFB50 regression with regard to training set of engine 2.

**Figure 26:**
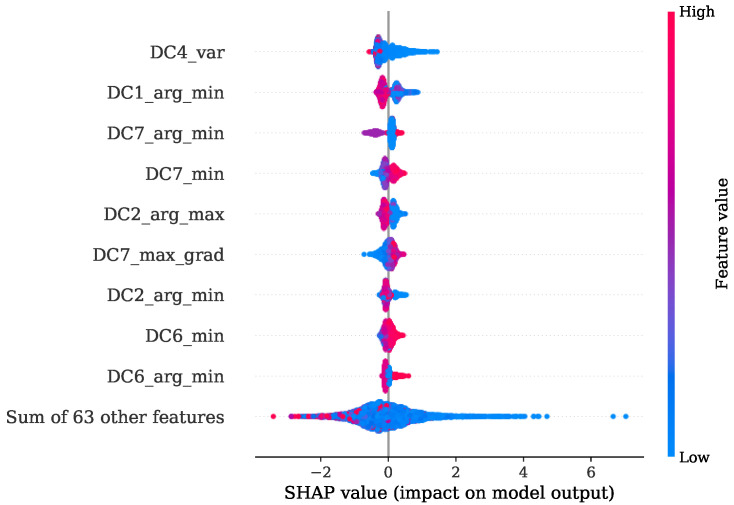
SHAP values summary of MFB50 regression with regard to training set of engine 2.

Comparing the FI based on MDI of Figure 25 and the SHAP evaluation of Figure 26 it can be observed that a good agreement is obtained. The feature contributing most were the variance of level 4, together with the location of minimum and maximum of levels 7, 1 and 2 respectively.

### 4.3. Summary

Table 3 presents an overview of all achieved results. Besides the experiments that were already shown in detail in Section 4.1 and Section 4.2, the results of regression tasks for MFB10 and MFB90 are presented. Moreover, the comparison method results are shown.

By looking at Table 3, it can be observed that the results obtained from engine 1 experiments have a higher error despite having a higher $R^{2}$ score. This can be explained with a different range of value distribution. By observing the point clouds of the regression plots from engine 1 shown in Figure 11 and Figure 12, it can be seen that values are ranging approximately from 50 to 200 bar. When compared with this range, the regression plots of engine 2 from Figure 15 and Figure 16 show a significantly narrow range with values from 100 to 170 bar. The same observation can be made by looking at the MFB50 regression plots from engine 1 data presented in Figure 19 and Figure 20, where values are ranging from approximately 3 to 28 °CA, while engine 2 experiments shown in Figure 23 and Figure 24 the values are ranging from 6 to 18 °CA. From this comparison, it can be seen how important it is to use various metrics. In this particular case, the $R^{2}$ score provided a misleading impression.

A comparison of the results from Table 3, which were obtained from the two applied methods, yielded a remarkable improvement that could be achieved by using the DWT. For all the experiments done, the comparison method with the time and the frequency features could not perform as well as the presented approach.

In the study of Posch et al. [7], results for the RMSE of PFP values of below 5 bar and the corresponding values for the RMSE of the PFP angular position of below 1.25 °CA were reported. By looking at Table 3, it can be seen that for the engine 1 test experiment, the RMSE value of PFP is 5.69 bar, whereas for the engine 2 test experiment, the obtained RMSE value of PFP is 3.7 bar. With regard to the angular position of the PFP, the MFB50 values shall be compared. From Table 3, it can be observed that all RMSE values of MFB50 are below 0.89 °CA. The performance of estimation of the MFB10 and the MFB90 parameters is worse compared to the MFB50 parameter. Nevertheless, with a MAE of 1.20 °CA for the worst scenario these results are still acceptable.

**Table 3:** Summary of regression results for all selected combustion parameters of introduced approach and comparison method.

	DWT + XGBoost											
	**Engine 1**						**Engine 2**					
	**Validation**			**Test**			**Validation**			**Test**		
**Target**	**RMSE**	**MAE**	***R*** **^2^**	**RMSE**	**MAE**	***R*** **^2^**	**RMSE**	**MAE**	***R*** **^2^**	**RMSE**	**MAE**	***R*** **^2^**
**PFP**	3.98	3.00	0.98	5.69	4.35	0.96	3.87	2.97	0.87	3.7	2.89	0.82
**MFB10**	1.38	1.09	0.83	1.43	1.14	0.77	0.84	0.66	0.53	0.82	0.65	0.48
**MFB50**	0.79	0.55	0.97	0.89	0.63	0.95	0.71	0.54	0.80	0.66	0.52	0.74
**MFB90**	1.42	1.05	0.96	1.43	1.09	0.79	1.63	1.20	0.91	1.42	1.10	0.74
	**Time/Frequency + XGBoost**											
	**Engine 1**						**Engine 2**					
	**Validation**			**Test**			**Validation**			**Test**		
**Target**	**RMSE**	**MAE**	***R*** **^2^**	**RMSE**	**MAE**	***R*** **^2^**	**RMSE**	**MAE**	***R*** **^2^**	**RMSE**	**MAE**	***R*** **^2^**
**PFP**	6.25	4.77	0.96	7.73	6.06	0.92	5.24	4.12	0.76	4.99	3.95	0.67
**MFB10**	1.70	1.34	0.73	1.90	1.50	0.59	0.97	0.76	0.39	0.96	0.76	0.30
**MFB50**	1.78	1.24	0.86	2.03	1.44	0.71	1.01	0.77	0.60	0.90	0.71	0.51
**MFB90**	2.60	1.88	0.86	3.01	2.17	0.69	1.91	1.47	0.63	1.82	1.40	0.56

## 5. Discussion

### 5.1. KS Position

The exact position and mounting of the KSs is a very delicate issue. Many studies exist showing promising methods with successful results, although the particular position of the sensor is not clearly presented. This is a hindrance concerning reproduction and comparison of these methods. Since physical conditions are often not identical from one engine block to another, a strategy is important to ensure comparable signal quality. One option is to position the sensor on an engine main bolt which is not in the proximity of the combustion chamber. These large dimensioned bolts are a structural requirement due to high loads and extreme physical conditions that occur inside the cylinders. This could be a promising approach, at least for most of the large single-cylinder gas engines, since these bolts have a machined surface and an appropriate drill included. No further machining is thus necessary. The method for estimating the combustion parameters accurately from the KS signal has to be precise and robust, even if the sensor and the events of interest inside the combustion chamber are not in proximity to each other. In this study it was shown that, even with the presence of such an extended transfer function of the engine block, a successful estimation of combustion parameters from the KS signal is possible. With the right signal-processing technique, which in the present case is DWT, it is possible to separate the useful information from interfering noise.

### 5.2. Towards a Theoretic Explanation of FI

After performing DWT on the KS signal a(ϕ), as well as on the in-cylinder PS p(ϕ), the results shown in Figure 9 are obtained. On looking at levels 6, 7 and 8, it can be seen that an approximately symmetrical characteristic of the KS signal a(ϕ) in blue and the pressure signal p(ϕ) in orange were obtained. This could be an indication for achieving an accurate estimation of the selected combustion parameters with DWT features.

The experiments for the PFP regression of Section 4.1 demonstrate that the crucial features were the RMS and variance features of DWT levels 1, 2 and 4 in combination with the location of the minimum from level 7. The implementation of the Haar motherwavlet constitutes a low-pass filter. Since, according to the Nyquist frequency, in lower levels of DWT a higher number of coefficients are received, it can be assumed that the energetic features will be more informative in comparison to higher levels with less coefficients. A valuable contribution of the RMS feature and the location of maximum was also found in the study of Siano et al. [11]. In this paper the authors low-pass filtered the KS signals and extracted these features for a non-linear regression task of the PFP and its related angular position.

In the specific task of PFP regression for the engine 1 data, the presented method has great potential for further simplification without losing significant performance by simply picking the most decisive features. This is also indicated by Figure 14, where it is illustrated that the contribution coming from the sum of all remaining 63 features is a disarranged point cloud of all colors represented. The second experiment regarding the PFP regression of engine data 2, on the other hand, showed a more spread FI which was also confirmed by the SHAP values from Figure 18. Here, further simplification by reducing the number of features would introduce higher losses in performance.

In Section 4.2, it is shown that for engine 1, the most informative feature was the maximum gradient from DWT level 7. This feature has mainly low-frequency proportions. Since the gradient of pressure constitutes the calculation of the MFB50 as shown in Equation (1), it is worth noticing this feature standing out. The location of minimum and maximum of level 7 is also found crucial in both training attempts for engine 1 and engine 2.

It would appear that all the proposed features are relevant for an accurate estimation of the chosen combustion parameters with only one exception which is the PFP of engine 1. Thus, a satisfying accuracy could be achieved, following the principle of ensemble learning in which many weak learners are used to improve the overall performance.

## 6. Conclusions

The investigation of representative datasets from two measurement campaigns of different engines yielded that, in both cases, the estimation of combustion parameters was successful. The proposed method proved to be able to separate useful information from noise with the signal-processing technique known as DWT. By extracting selected statistical features from the coefficients received by the DWT and using these features as input for XGBoost regression models an estimation of the selected combustion parameters PFP, MFB10, MFB50 and MFB90 was obtained.

The PFP regression reached an MAE of 2.89 bar for the validation set of engine 1 and an MAE of 4.35 bar for unseen OPs of the corresponding test set. Experiments of the second dataset from engine 2 revealed an MAE of 2.97 bar on the validation set and an MAE of 2.89 bar on unseen OPs from the test set. The MFB50 regression of engine 1 data performed with an MAE of 0.55 °CA on validation set and an MAE of 0.63 °CA for unseen OPs of the test set. In comparison, an MAE of 0.54 °CA for validation and an MAE of 0.52 °CA for testing were obtained from engine 2 data.

The presented approach shows the promising potential of replacing the expensive and not-durable in-cylinder PS with a low-cost KS. This method has the capability of dealing with KS positions not necessarily located in proximity of the combustion chamber. With this property, no additional machining steps are required, and the mounting of the sensors is easily performed. Furthermore, only one source of information alone the signals from the KS is sufficient to achieve a precise estimation of the desired combustion parameters. A further advantage is that no calibration of filters is necessary for the successful implementation of the presented method. Since the sources of useful information were identified with the FI based on MDI and confirmed by the SHAP value analysis, the potential of reducing the complexity for individual regression tasks by preserving most of the efficiency is given.

Future research steps constitute the collection and comparison of various methods from the literature for in-cylinder pressure reconstruction and estimation of combustion parameters using KS signals. Furthermore, the implementation of the presented approach on an ECU for a closed-loop control strategy will be investigated.

## Figures and Tables

**Table 1:** PFP regression results for validation set of engine 1 with various mother wavelets.

Metric	Haar	Db4	Sym4	Coif6
MAE	3.00	3.97	3.94	4.31
RMSE	3.98	5.12	5.11	5.57
$R^{2}$	0.98	0.97	0.97	0.97

**Table 2:** Selection of Features.

Description	Name	Equations
1. Index of minimum	arg_min	$\phi_{min}=argmin\left(c\right)$
2. Index of maximum	arg_max	$\phi_{max}=argmax\left(c\right)$
3. Variance	var	$\sigma^{2}=\sum_{i=1}^{n}(\frac{{\left(c_{i}-\bar{c}\right)}^{2}}{n-1}$)
4. Maximum	max	$c_{max}=max\left(c\right)$
5. Minimum	min	$c_{min}=min\left(c\right)$
6. Maximum gradient	max_grad	$\frac{\partial c}{\partial \phi }{|}_{max}=max\left(\frac{\partial c}{\partial \phi }\right)$
7. Mean difference	mean_diff	$\bar{d}=\bar{c_{i+1}-c_{i}}$
8. Root mean square	rms	$c_{rms}=\frac{\sqrt{\sum_{i=1}^{n}{c_{i}}^{2}}}{n}$

## Abbreviations

The following abbreviations are used in this manuscript:

ANN	Artificial neural network
AC	Approximation coefficients
CA	Crank angle
CWT	Continuous wavelet transfrom
DC	Detailed coefficients
DWT	Discrete wavelet transform
ECU	Engine control unit
FI	Feature importance
IMEP	Indicated mean effective pressure
KS	Knock sensor
MAE	Mean absolute error
MDI	Mean decrease in impurity
MFB10	Mass fraction burned 10%
MFB50	Mass fraction burned 50%
MFB90	Mass fraction burned 90%
OPs	Operating points
PFP	Peak firing pressure
PS	Pressure sensor
RMSE	Root mean square error
RMS	Root mean square
$R^{2}$	Coefficient of determination
SCE	Single cylinder engine
SHAP	Shapley additive explanations
XGBoost	Extreme gradient boosting
